# Response to: American College of Radiology Appropriateness Criteria®: A bibliometric analysis of panel members

**DOI:** 10.1186/s13244-023-01457-y

**Published:** 2023-07-19

**Authors:** Mark E. Lockhart, David B. Larson, William T. Thorwarth

**Affiliations:** 1 University of Alabama at Birmingham, 1720 University Blvd, Birmingham, AL 35294 USA; 2 Department of Radiology, Stanford University School of Medicine, 453 Quarry Rd, Mail Code 5659, Stanford, CA 94305 USA; 3 American College of Radiology, 1892 Preston White Dr, Reston, VA 20191 USA

We thank the journal for this opportunity to respond to “American College of Radiology Appropriateness Criteria®: A Bibliometric Analysis of Panel Members” [1] and provide clarification of our panel selection process [2]. We also welcome the opportunity to better explain the purpose of the Appropriateness Criteria (ACR AC) and process by which they are generated.

ACR AC are developed to assist referring clinical providers to order the appropriate imaging examination by synthesizing current medical literature into recommendations for specific clinical conditions. The program depends on two key facets to achieve that end: (1) diverse, multi-disciplinary teams with a range of types of experience and expertise, utilizing (2) a rigorous systematic process to evaluate the literature and agree on recommendations.

The main issue raised by the authors is what they perceive to be a deficiency in scholarly expertise, which they measure as number of prior publications in the area relevant to the topic. The ACR AC empaneling process seeks to recruit unbiased experts in specific technical areas who are willing to donate their time while managing potential conflicts of interest [2]. This process takes a broader view of expertise than those of the authors, consistent with the Institute of Medicine [3] methodology. For example, in addition to relevant publications, we consider years in practice, areas of clinical focus, awards or recognition by peers, fellowship recognition in professional societies, invitations to proctor for certifying exams, invited course leads or presentations at scientific or educational meetings, and leadership roles in relevant societies.

Additionally, while contributions to the published literature provide an important background in developing appropriateness criteria, the concept of appropriateness must also incorporate the practical, sometimes messy, realities of varying local practice contexts that can never be fully reflected in the literature. For this reason, ACR AC panels are intentionally composed of individuals with diverse backgrounds and types of expertise. Such perspectives provide an important balance to those focused on conducting and publishing research, who often spend less time in the clinical environment. In short, we would consider it a weakness if all AC panels only included experts with elite academic credentials.

Our panels of volunteer experts are recruited for a set period of time during which they participate in the development of all the papers in their general area of practice (such as musculoskeletal imaging). Their broader clinical expertise in the area, rather than focused expertise on just one particular topic, allows for a broader input into each topic and a diversity of viewpoints. These panels function as a cohesive group, with intentional redundancies and overlap to faithfully reflect the evidence where it exists and to ensure thoughtful discussions on points where the evidence is less clear. There is ample opportunity for each panelist to weigh in, and no single voice overpowers the collective view. We continually monitor the composition of panels through self-assessment, conflict of interest forms, practice demographics, and geography to ensure wide representation of perspectives and expertise. Areas of expertise represented on panels include clinical trial design, evidence-based medicine, clinical guideline development, quality improvement, and statistical analysis.

In recruiting non-radiologist referring providers, we rely on national societies to identify experts who best represent practice in an area. The extensive participation by non-radiologists who represent the primary audience of physicians who order imaging is a particular strength of the AC program; these experts bring clinical input to the process to ensure that the guidelines developed are meaningful and helpful for image ordering.

All the experts on the panels are volunteers. Our society is incredibly fortunate to recruit hundreds of highly capable and motivated medical experts who provide their services without reimbursement for the betterment of medical practice. Their cumulative contribution constitutes a unique gift to the entire world, which is reinforced by the international attention the program draws, including from these authors. Along with this remarkable volunteer effort come a few challenges. For example, we acknowledge the authors’ criticism that most of the ACR AC panel experts are from academic teaching institutions. This is due to the simple fact that academic radiologists tend to have more protected time to pursue such volunteer activities, though we continually strive to recruit private practice physicians who are able to devote the extensive time necessary for this volunteer effort.

The authors criticize the program’s focus on imaging efficacy rather than effectiveness or outcomes. We agree with the authors that this is a limitation to the extent that directly linking imaging to clinical outcomes constitutes a challenge inherent to diagnostics broadly given that diagnostic assessments inform medical and surgical management, which are more directly related to outcomes.

The authors raise concerns about the strength of evidence methodology upon which the AC are based. The methodology as translated from GRADE is described publicly [1], and each AC article contains an evidence table assessing the strength of evidence. The evidence table, literature search, and appendix for this topic are available online with the full AC topic list [4]. The appendix includes the strength of evidence assessment and the final rating round tabulations for each recommendation. As the authors note, the entire methodology is also posted online on the main ACR AC landing webpage [2, 5–9] to bolster the transparency of the process.

The authors mention that not all available evidence is included in all AC. Our panels conduct systematic searches, but we acknowledge that they likely miss some relevant articles, as any expert might. We continually accept and welcome feedback on our review site [10], available for anyone to provide information and thoughts on the recommendations, clinical scenarios, evidence, or any aspect of the documents. All such feedback is thoughtfully considered in the subsequent round of AC review.

In conclusion, we believe that the methodologic concepts used for the ACR AC are well-suited for the program’s goals and consistent with accepted guideline development practices that are used across the United States and internationally. While the program can always be improved, we have confidence in both the qualifications of the volunteer experts as well as the AC generation process to guide providers in the complex, dynamic, and highly context-dependent environments of real-world medical practice.

## Data Availability

There are no data used in this manuscript. All references used are cited and publicly available.
